# Supplementary material for: The Integrity of α-β-α Sandwich Conformation Is Essential for a Novel Adjuvant TFPR1 to Maintain Its Adjuvanticity
Source: Biomolecules. 2019 Dec 12;9(12):869. doi: 10.3390/biom9120869 (PMC6995627; doi:10.3390/biom9120869)
Supplement: Supplementary file 1 [file biomolecules-09-00869-s001.pdf]

Supplementary Materials

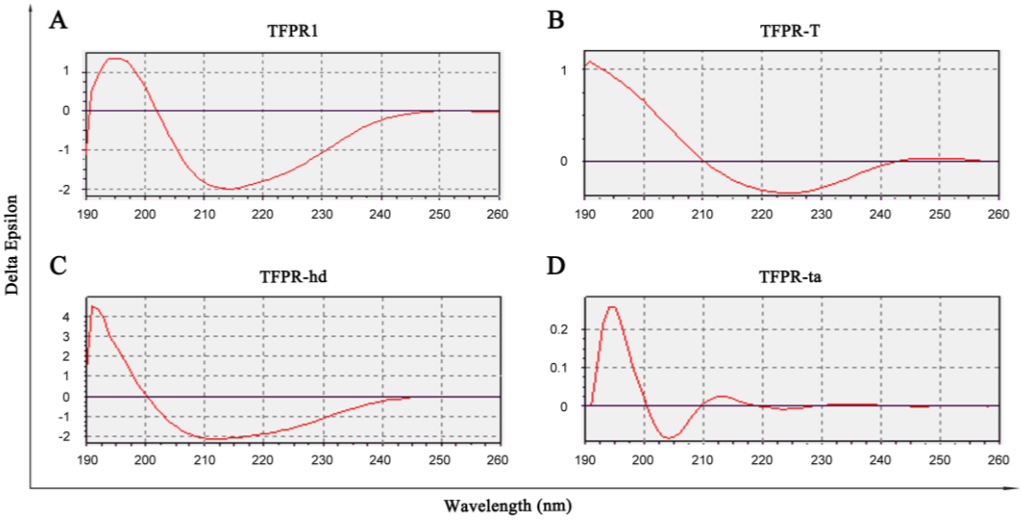

Figure S1. CD-spectrum of the four proteins TFPR1, TFPR-T, TFPR-hd and TFPR-ta. Concentration of each protein was adjusted to 0.4 μg/μL using PBS; 200 μL of protein solution was added to the cuvette, and absorption spectrum at wavelength of 190-260 nm was measured using Circular Dichroism (CD) spectrometer (Chirascan, Applied Photophysics Ltd, England), PBS was used as background; finally, each secondary structure content including α-helix, anti-parallel and parallel β-sheet, β turn and random coil were analyzed using CDNN software (Gerald Böhm, Magdeburg). (A) TFPR1, (B) TFPR-T, (C) TFPR-hd and (D) TFPR-ta. The results shown in these figures have deducted the background of PBS (Fig. S2), in which each protein was dissolved.

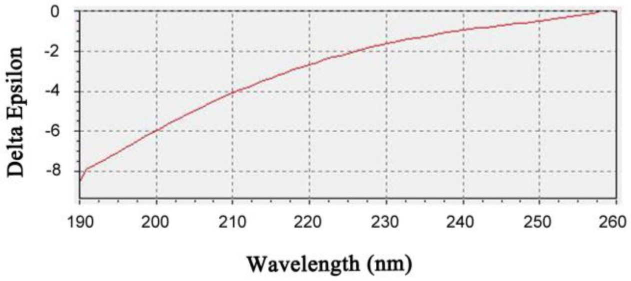

Figure S2. Spectrum analysis of PBS as background control by CD Spectrometer. PBS was used as background control of the CD-spectrum analysis of the four proteins, 200 μL of PBS was added to the cuvette, and absorption spectrum at wavelength of 190-260 nm was measured using Circular Dichroism (CD) spectrometer (Chirascan, Applied Photophysics Ltd, England).
